# Unlabeled Far‐Field Deeply Subwavelength Topological Microscopy (DSTM)

**DOI:** 10.1002/advs.202002886

**Published:** 2020-11-17

**Authors:** Tanchao Pu, Jun‐Yu Ou, Vassili Savinov, Guanghui Yuan, Nikitas Papasimakis, Nikolay I. Zheludev

**Affiliations:** ^1^ Optoelectronics Research Centre and Centre for Photonic Metamaterials University of Southampton Southampton SO17 1BJ UK; ^2^ Centre for Disruptive Photonic Technologies The Photonics Institute School of Physical and Mathematical Sciences Nanyang Technological University Singapore 637371 Singapore

**Keywords:** machine learning, microscopy, superoscillations, superresolution, unlabeled

## Abstract

A nonintrusive far‐field optical microscopy resolving structures at the nanometer scale would revolutionize biomedicine and nanotechnology but is not yet available. Here, a new type of microscopy is introduced, which reveals the fine structure of an object through its far‐field scattering pattern under illumination with light containing deeply subwavelength singularity features. The object is reconstructed by a neural network trained on a large number of scattering events. In numerical experiments on imaging of a dimer, resolving powers better than *λ*/200, i.e., two orders of magnitude beyond the conventional “diffraction limit” of *λ*/2, are demonstrated. It is shown that imaging is tolerant to noise and is achievable with low dynamic range light intensity detectors. Proof‐of‐principle experimental confirmation of DSTM is provided with a training set of small size, yet sufficient to achieve resolution five‐fold better than the diffraction limit. In principle, deep learning reconstruction can be extended to objects of random shape and shall be particularly efficient in microscopy of a priori known shapes, such as those found in routine tasks of machine vision, smart manufacturing, and particle counting for life sciences applications.

## Graphical abstract

Nonintrusive, label‐free, far‐field optical microscopy is introduced based on illumination of an object with topologically structured light that contains multiple deeply subwavelength singularity features. This new type of optical microscopy allows to resolve the fine structure of an object with resolution that can exceed the conventional diffraction limit by two orders of magnitude.

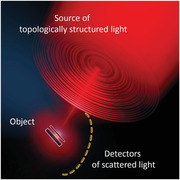

## 1 Introduction

The development of label‐free far‐field superresolution microscopy, beyond the half‐wavelength limit of the conventional microscope, remains one of the main challenges for science and technology. Indeed, the ability to image at the nanometer scale using visible light will open unprecedented opportunities in the study of biochemical, biomedical, and material sciences, as well as nanotechnology. However, despite persistent research efforts, deep subwavelength resolution is only possible using techniques, such as STED^[^ ^2^ ^]^ and SMLM / STORM^[^ ^3^, ^4^ ^]^ that require labelling of samples with luminescent material that is not acceptable for many in vivo biomedical applications due to toxicity and label introduction complexity and is not suitable for nanotechnology applications (e.g., imaging of semiconductor chips). Very recently, it was demonstrated that far‐field, noncontact, label‐free, optical, high‐resolution imaging can be achieved by analyzing intensity patterns of light scattered by the object using artificial intelligence.^[^ ^5^, ^6^ ^]^ Here we demonstrate by virtue of numerical and proof‐of‐principle real‐life experiments that further improvement in resolution can be achieved by illuminating the object with topologically structured light. The improved resolution results from the interactions of the object's fine features with singularities of highly structured topological light. We term our method deeply subwavelength topological microscopy (DSTM).

To justify the term of DSTM, we note that broadly speaking, in the contemporary context, “imaging is the representation or reproduction of an object's form.”^[^ ^7^ ^]^ Historically, for centuries imaging was a technique for representing an object's form by creating a light pattern resembling the object, in the same way that a conventional microscope creates a light pattern on the retina of the observer's eye or a screen. The proliferation of computers and image processing techniques has often replaced such light patterns by patterns on the computer screen, or data stored in the computer memory. This is now common practice in modern optical imaging techniques, such as confocal imaging, SNOM, STED, as well as for most electron‐beam imaging techniques. We argue that the technique described in this work is a computer‐enabled imaging technique that provides a comprehensive representation of the object's form including all its dimensions and, in principle, allows full reconstruction of its shape. Our technique is also a form of microscopy according to the common definition of microscopy as “the technical field of using microscopes to view objects …that cannot be seen with the naked eye (objects that are not within the resolution range of the normal eye).”^[^ ^8^ ^]^

Recent microinterferometric experiments^[^ ^9^ ^]^ confirmed the theoretical observations^[^ ^10^ ^]^ that complex coherent optical fields contain highly localized intensity hotspots and zones of energy backflow. They also revealed that near topological singularities in such optical fields, the phase varies on a distance orders of magnitude smaller than the wavelength of light. Such optical fields with rapid phase variation are known as superoscillatory fields and can be generated through interference of multiple waves diffracted on a complex grating^[^ ^11^ ^]^ or purposely designed masks.^[^ ^12^ ^]^ Here, we show that DSTM can reveal the fine subwavelength structure of an object through the recording of intensity profiles of a number of far‐field scattering patterns under superoscillatory illumination. Our approach exploits the significant changes of the far‐field patterns of scattered light that take place when the deeply subwavelength features of the object overlap with the rapid spatial variations of the illuminating topological field.

In contrast to a conventional microscope that forms the image of the object in a single exposure and is limited in resolution at about half wavelength of the light used for illuminating the object, prior to imaging DSTM requires a training process, which involves advanced processing of multiple scattering field patterns. We show that a convolutional neural network trained on a large number of scattering events can reliably retrieve information about the object with deeply subwavelength resolution. Moreover, our analysis shows that the high resolution of our microscopy technique is tolerant to detector noise and is achievable with low dynamic range light intensity sensors.

A direct reconstruction of an imaged object is possible if the intensity and phase of the scattered field is known on a closed surface encompassing the object (Kirchhoff–Helmholtz integral). Recently developed monolithic optical microinterferometry^[^ ^9^ ^]^ can, in principle, detect the phase of the field everywhere around an isolated scattering object, but the technique is extremely challenging for routine microscopy.^[^ ^13^ ^]^ Although the field intensity of scattered light is much easier to measure, as no interferometry is required, image reconstruction from only the intensity profiles is an ill‐posed inverse problem.^[^ ^13^ ^]^

Interference‐based methods that allow the retrieval of partial phase information have been shown to increase the resolution of low numerical aperture imaging systems.^[^ ^14^ ^]^ Different iterative feedback algorithms have been developed enabling the reconstruction of an image from intensity of scattering patterns of optical, deep UV and X‐ray radiation with resolution essentially limited by the wavelength of the illuminating light in most cases,^[^ ^15^, ^16^ ^]^ and around 5‐times higher when compressed sensing techniques for imaging sparse objects are used.^[^ ^17^, ^18^ ^]^ Artificial intelligence methods have been used to improve conventional machine vision^[^ ^19^, ^20^ ^]^ and microscopy^[^ ^21^, ^22^ ^]^ focusing on reducing both the acquisition time and light intensity required for imaging labeled samples. However, opportunities that arise from using deeply subwavelength features of topologically structured light have not yet been explored.

The numerical and proof‐of‐principle experiments reported here show that label‐free imaging with deeply subwavelength resolution is possible by detecting only the intensity profile of the scattered light. We also show that illumination with topological light gives access to higher resolution than conventional plane wave illumination. We reconstruct the spatial dimensions of the object from the intensity profiles of scattered light with a deep learning neural network trained on a large number of scattering events. We demonstrate DSTM by imaging of a dimer, a pair of randomly positioned subwavelength particles of arbitrary size and separated by arbitrary distance, an important task that appears often in bioimaging and nanotechnology (e.g., cell division).

## 2 Results

**Figure 1:**
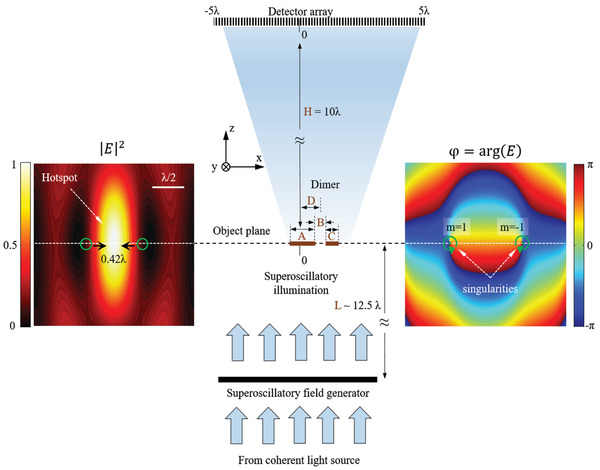
Deeply subwavelength topological microscopy (DSTM) schematic. The imaged object (a dimer *A*–*B*–*C*) is illuminated with a superoscillatory light field. The intensity profile of the diffraction pattern resulting from scattering of the superoscillatory light field on the imaged object is detected by the detector array. A number of different diffraction patterns are recorded when the illuminating field is scanned against the object. Left and right panels show maps of intensity and phase profiles of the illuminating field and indicate the presence of hotspots and phase singularities, where *m* indicates the winding number of the singularity.

In our modelling microscopy experiments (see **Figure** **1**), we consider 1D imaging of a dimer consisting of two totally absorbing, nonscattering elements of widths A and C with gap B between them. The dimer's location from the center of the object plane is represented by distance D. The scattered light is detected by an intensity detector array that is placed at a distance of *H* *=*10*λ* from the object plane, over its center. Here *λ* is the wavelength of the free‐space radiation used in the modeling. We assume that the detector array is 10*λ* long. Since the scattered field reaching the detector array is formed by free‐space propagating waves, in a real experiment it can be imaged by a conventional lens at any magnification without loss of resolution, by simply adjusting the magnification to ensure that the detector pixels are smaller than the required resolution, as has already been realized experimentally.^[^ ^9^ ^]^ We therefore assume that the detector array can image the intensity profile of the diffracted/scattered light without limitations to spatial resolution and conduct our modelling for a detector array containing five thousand pixels. As we will discuss below, DSTM is remarkably resilient to limitations in the detector's dynamic range and the presence of noise.

We consider two closely related situations, where the position *D* of the dimer at the imaging plane is either known or unknown. We assume that the dimer with unknown position is located anywhere within a chosen interval. In the former case, the microscopy returns the dimensions of A, B, C of the dimer, whereas in the latter the position D is returned in addition to the dimer dimensions. We use topological light illumination in the form of a superoscillatory wavefront generated by a planar Pancharatnam–Berry phase metasurface that was developed in ref. ^[^ ^1^ ^]^, which creates a superoscillatory subwavelength hotspot at a distance *L* = 12.5*λ* from the plane of the metasurface (superoscillatory field generator). Here, we consider only the central part of the superoscillatory field, where the hotspot is flanked by singularities and zones of high phase gradient (see Figure 1). Similar patterns can be synthesized by spatial light modulators and this will be used in the proof‐of‐principle real experiment described below. For comparison, we also used plane wave illumination. The scattered field of the dimer is recorded at eleven positions by moving the superoscillatory hotspot across the object in intervals of *λ*/5, from the −*λ* position to the +*λ* position in the object plane. The corresponding diffraction patterns are then recorded, and the full set of diffraction patterns is analyzed by a convolutional neural network^[^ ^23^ ^]^ trained with the Adam stochastic optimization method^[^ ^24^ ^]^ (see Supporting Information). The training dataset contained 20000 samples of dimers with known dimensions. It was generated by creating dimers of random sizes and placing them on the object plane with the dimer center coordinate D randomly chosen in the interval from –*λ*/2 to *λ*/2 (in the case of unknown dimer position). The widths of the dimer components (A and C) and the gap between them (B) were independently and randomly chosen between 0.002*λ* and *λ*. The diffraction pattern on the detector array was then calculated by the Fourier propagation method for the transverse component of the electric field.

**Figure 2:**
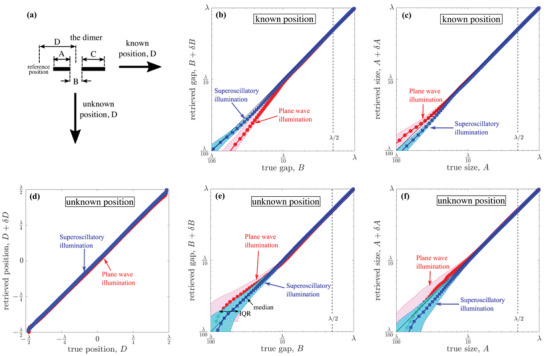
Deeply subwavelength topological microscopy of a dimer. a) The dimer consists of two elements with different sizes A and C separated by a gap (edge‐to‐edge) B. It is positioned in the object plane at distance D from the *x* = 0 points of the object plane (see Figure 1). Two different regimes are presented, where the dimer position is either b,c) known (fixed at D = 0), or d–f) unknown. b,c) The retrieved values of B and A presented against their actual values, when D is known. Solid blue and red lines correspond to the median of the true values under superoscillatory (blue squares) and plane wave illumination (red circles), while the red and blue colored bands indicate the corresponding interquartile (IQR) ranges (see also the Supporting Information). In the case of unknown position, panels (d)–(f) show the retrieved values of D, B, and A presented against their actual values. Retrieved values for size C are similar to size A.

**Figure 3:**
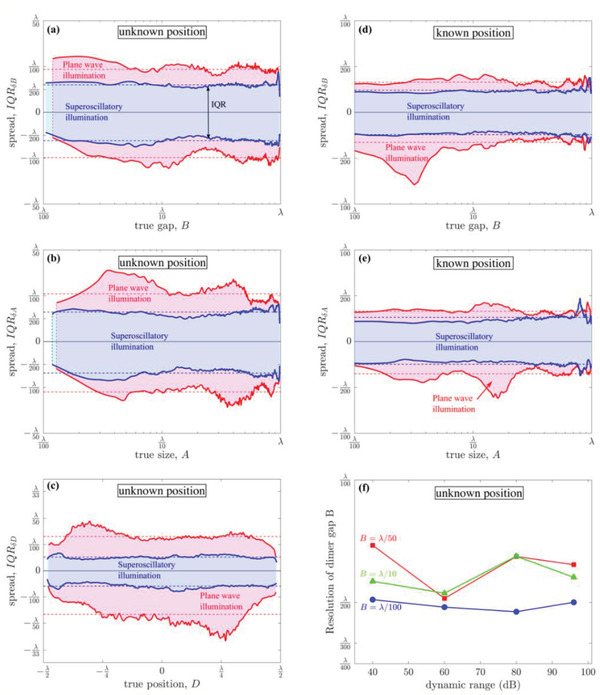
Resolution of the deeply subwavelength topological microscopy. IQRs of measured values of the dimer dimensions, a,d) gap, B, b,e) element size, A, and c) position, D, during numerical imaging experiments with a–c) unknown and d–e) known dimer position. Red and blue colored regions correspond to plane wave and superoscillatory illumination, respectively, while red and blue solid lines mark the first and the third quartiles of the corresponding error distributions. The horizontal dotted lines indicate the average value of the IQRs over the range of the true values of the respective dimension. The vertical dotted lines in panels (a) and (b) indicate the geometric dimension's true value below which the network returns predominantly negative, nonphysical values. f) Dependence of resolution (in dimer gap B) as a function of the dynamic range of the photodetector.

The results of our numerical microscopy experiments are presented in **Figures** **2** and **3**. To evaluate the resolution of the method, we imaged ∼7 × 10^5^ dimers with randomly selected dimensions (0.01λ ≤ *A*,*B*,*C* ≤ λ) and position (−λ/2 ≤ *D* ≤ λ/2). We analyzed the results statistically by grouping the retrieved values for each of the dimer parameters, *A*, *B*, *C*, and *D*, in bins each containing 5000 results and calculating the median and spread within each bin.

We found that the dimensions of the dimer and its position can be retrieved with deep subwavelength resolution. Indeed, on Figure 2 the solid red and blue lines correspond to the median of the true values as a function of the retrieved value, whereas the black solid line is the bisector of the first quadrant (*y* = *x*) representing perfect agreement between true and retrieved values. A departure of the median from the bisector represents a systematic bias in the retrieval process. When the position of the dimer is known, we obtain remarkably accurate retrieval of all dimensions both for plane wave (red lines) and topological superoscillatory illumination (blue lines), with the systematic bias of ≈*λ*/100 or smaller. Here, superoscillatory illumination gives similar results to plane wave illumination for the size of dimer's element A, but provides over a factor of ×2 smaller systematic bias for dimer gap B. When the dimer position is a priori unknown, the systematic bias increases but remains sub‐*λ*/100 with superoscillatory illumination still giving better results for retrieval of dimer gap B and position D than plane wave illumination.

The deeply subwavelength topological microscopy reported here retrieves the dimer's geometric dimensions probabilistically. Thus, we define as the resolution of DSTM the spread of retrieved values around the real value as quantified by the interquartile range (IQR), which indicates the interval within which 50% of the retrieved values are found (see Figure 3). Since IQR does not vary significantly with the dimensions of the dimer, we use its mean value as the method's resolution. Remarkably, in the case of known position and superoscillatory illumination, the resolution of the imaging process exceeds *λ*/200 for all dimer dimensions. When the position of the dimer is not known, the resolution decreases to ≈*λ*/80 for superoscillatory illumination. In both cases, superoscillatory illumination provides a resolution enhancement of >50% over plane wave illumination (see the Supporting Information).

The results presented in Figure 3 were obtained by using the field amplitude of the diffracted pattern resolved with 16‐bit precision corresponding to a dynamic range of 96 dB. Here the dynamic range is defined as 10·log10(*I*_max_/*I*_min_), where *I*_min_ and *I*_max_ are the minimum and maximum intensity levels that can be recorded. Although such dynamic range is achievable with high‐quality photodetectors, the resolution of the method is weakly dependent on the detector's dynamic range. To illustrate this, the detector's dynamic range was deliberately reduced by rounding readings to lower values (Figure 3f). Nevertheless, resolution at the *λ*/100 scale is achieved even for 40 dB dynamic range, whereas typical photodiode values are well above the 60 dB level.

Apart from the dynamic range of the detectors, resolution will also be constrained by noise at the detector. However, our results (see Figure S3 in the Supporting Information) indicate a remarkable resilience of the method: even in the case of 5% random noise (a very high value for high quality electro‐optical systems), a dimer can be imaged at a resolution of ≈*λ*/71 for the element size, ≈*λ*/77 for the gap, and ≈*λ*/92 for the position.

In practical terms, the main challenge in the experimental implementation of DSTM would be in creating reliable and trustworthy training sets for deep learning. Such datasets can be either virtual or physical. The computer generated training dataset of imaged objects and their scattering patterns can be rapidly and accurately generated by computing scattering profiles on a random set of virtual training objects. Here the main challenge is to make the computer model congruent with the physical realization of the microscope to allow adequate imaging of the real object. Alternatively, a physical dataset can be created by nanofabrication of a number of real scattering elements by electron beam lithography or focused ion beam milling followed by recording of their real scattering patterns in the physical imaging microscope. Generating a physical set is labor‐intensive, but such a set will be naturally congruent with the imaging microscope. The choice of the training dataset (physical or virtual) shall be informed by the desired resolution and complexity of the microscope optical tract. In fact, we expect that the required dataset size will scale with the complexity and dimensionality of the imaging target and will depend on the *a priori* available information. Indeed, objects of higher complexity or dimensionality (e.g., from *1D* to *2D* or *3D*) will be described by a larger number of geometric dimensions and thus, we argue that increasingly larger training datasets will be required. For instance, if in dimer microscopy the required resolution is only *λ*/10, a training set involving only ≈100 objects is needed, while microscopy with resolution exceeding *λ*/200 may require training datasets comprising scattering events on tens of thousands objects (see Figure S4 in the Supporting Information). Depending on the size of the training dataset and the complexity of the imaging target training of the network may take considerable time (one hour in a multi‐GPU workstation in our demo numerical experiments described above). However, once trained, image reconstruction is possible with video frame rates.

We demonstrate the practicality of using physical datasets in DSTM by proof‐of principle experiments on imaging of dimer slits fabricated in an opaque metallic film by focused ion beam milling. Each dimer comprises a pair of nanometer scale slits of unknown width in the range 0.26*λ*–1.10*λ* and spacing between them in the range 0.17*λ*–0.94*λ* (see the Supporting Information for experimental details).

For the experiment we use the framework of a conventional dual microscope equipped with a sample piezo nanopositioning stage with 100 nm resolution. The dimer is placed on the object plane of the imaging apparatus and illuminated with coherent laser light at the wavelength *λ* = 488nm. Light diffracted from the dimer is then imaged at a distance of *H* = 2*λ* from the object plane by a high‐numerical lens (NA = 0.95) and a 2.1‐megapixel sCMOS array. Since the diffracted field reaching the detector array is formed by free‐space propagating waves, it can be imaged at any magnification without loss of resolution by adjusting the magnification level necessary to ensure that the detector pixels are smaller than the required resolution. The imaging system of our apparatus had magnification of 400 corresponding to the pixel size of 12.6 nm on the image plane.

We used two types of illumination created by a computer‐controlled wavefront synthesizer system based on spatial light modulators.^[^ ^25^ ^]^ In the DSTM modality, we used topologically structured light illumination consisting of a superoscillatory subdiffraction hotspot surrounded by a halo of concentric rings. Results obtained with topologically structured illumination were compared to Gaussian profile illumination with a focus larger than the dimer. In the DSTM regime, the measurements were performed at 21 different positions of the superoscillatory hotspot on the dimer, by gradually shifting the hotspot with steps of *λ*/4.9 perpendicular to the slit direction in the object plane using the piezo nanopositioning stage.

The full experimental set consisted of 144 dimers, of which 115 dimers were used for neural network training, 14 dimers for validation, and 15 dimers for our test imaging experiments. Upon fabrication all dimers were measured to nanometer precision with a scanning electron microscope. The diffraction patterns from the set of 115 dimers were recorded in the imaging apparatus and together with their dimensions formed the neural network training set.

**Figure 4:**
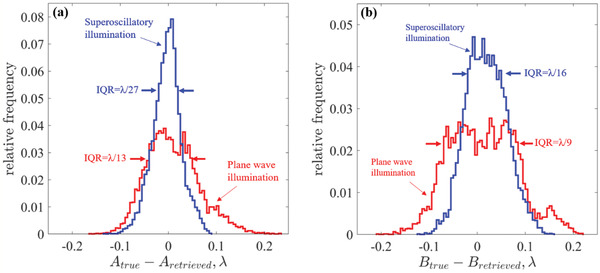
DSTM proof‐of‐principle experiment. a,b) The statistical distribution of the retrieved results for the width of the a) dimer component A and b) the dimer gap B presented as the difference between the retrieved value and the true value as measured with scanning electron microscope. Blue lines correspond to results obtained with superoscillatory illumination, while red lines correspond to broad Gaussian illumination. The histogram is calculated from retrieved parameters corresponding to 500 different realizations of the neural network.

Upon completion of the training, the apparatus was ready for imaging dimers of unknown size. We repeated the training process for 500 different realizations of the neural network and present the corresponding average values in **Figure** **4**a,b. We observed that:

1) The gap, B, and the widths of the slits, A and C, are retrieved with subdiffraction resolution.
2) The resolution of retrieving the size of the gap and the width of the slits is higher for topologically structured illumination than for Gaussian profile illumination.
3) Even a small training set of 115 dimers has been sufficient to achieve deeply subwavelength resolution of *λ*/16 for the gap and *λ*/27 for the slit width (as quantified by the by the interquartile range of the distribution) using topologically structured light illumination. At the same time Gaussian illumination only returned resolution of *λ*/9 for the gap and *λ*/13 for the slit width.

**Figure 5:**
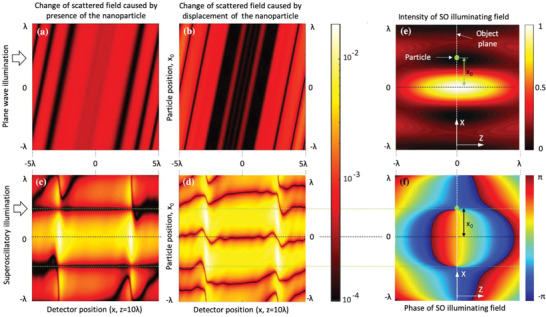
Sensitivity of far‐field intensity patterns on the presence and position of an absorbing nanoparticle that is *λ*/1000 in size. a,c) Normalized change of the scattered field intensity profile caused by presence of the nanoparticle. b,d) Normalized change of the scattered field intensity profile caused by shifting the nanoparticle in steps of *λ*/2000 along *the x* direction. Panels (a) and (b) correspond to plane wave illumination; panels (c) and (d) illustrate illumination with a superoscillatory field. Maps (e) and (f) show intensity and phase profiles of the illuminating superoscillatory field, where light propagates along the positive *z*‐axis.

We argue that the observed factor of ≈x2 improvement of the resolution with topologically structured light illumination in comparison with Gaussian illumination reported in this proof‐of‐principle experiment can be improved much further by increasing the number of different positions of the superoscillatory hotspot on the object (the step size in the current experiment is *λ*/4.9 = 100 nm). The fine scanning will take full advantage of strong variations of the scattered field when the structural features of the object overlap with that of the illuminating field (see **Figure** **5** and discussion below). This would require a more precise sample positioning system and improved mechanical stability of the imaging apparatus at the level of AFM/STM instruments. Nevertheless, our results unambiguously demonstrate the resolution advantages that topologically structured illumination delivers.

## 3 Discussion

The numerical DSTM experiments performed with large training sets and the real proof‐of‐principle experiments conducted with a small training set confirmed that artificial intelligence enabled retrieval of the imaging target dimensions from the intensity patterns of the scattered field delivers deeply subwavelength resolution. The deeply subwavelength level of resolution reported here significantly exceeds the Abbe “diffraction limit” of resolution (≈*λ*/2). We have also observed, both in numerical and real‐life experiments, that using topologically structured illumination increases the resolution of microscopy. We argue that several factors contribute to this improvement:

1) Recording of multiple scattering patterns during the training process and imaging provides much more information on the imaged object for the retrieval process than what is available in the lens‐generated single image for which the Abbe limit has been derived.
2) The deep learning process involving a neural network trained on a large dataset creates a powerful and accurate deconvolution mechanism without using explicit information on the phase of the detected signals.
3) Sparsity of the object and prior knowledge about the object (dimer of unknown size and location) help the retrieval process, similarly to how sparsity helps “blind” compressed sensing techniques.^[^ ^17^ ^]^
4) Topological illumination ensures much higher sensitivity of the pattern of scattered light to small features of the imaged object than conventional illumination.

The last argument requires a more detailed comment. Superoscillatory fields contain zones of rapid phase gradients and high local wave vectors. This could explain the high spatial resolution through Fourier connection between spatial and reciprocal space. Although this fact is reassuring, its full implications are difficult to analyze in the context of the multiple exposures and the neural network deconvolution used in the DSTM technique. Instead, in Figure 5 we illustrate the sensitivity of the scattered field pattern on placing a small absorbing nanoparticle in the illuminating topological field. The nanoparticle, only *λ*/1000 in size, is positioned on the object plane at coordinate *x*_0_ (see Figures 1 and 5e,f) and illuminated with coherent light of wavelength *λ*. The intensity of the scattered light is detected at a distance *H* = 10*λ* from the nanoparticle at points with coordinates (*x*, *z* = 10*λ*). Maps (a) and (c) in Figure 5 illustrate sensitivity of scattering to the presence of the particle in the illuminating field for plane wave and superoscillatory illumination, respectively. They show the normalized change of the intensity of scattered light (colormap, logarithmic scale) as a function of the particle position *x*_0_ on the object plane and the detector's coordinate *x*. Maps (b) and (d) illustrate sensitivity of scattering to small displacements of the particle. They show the normalized change of the scattered field intensity (colormap, logarithmic scale) on displacing the particle with step of *λ*/2000 along the object plane with the particle initially located at *x*_0_. From Figure 5, it follows that scattering of the superoscillatory field is two to three orders of magnitude more sensitive than in the case of plane wave illumination to the presence and repositioning of the nanoparticle, which we attribute to the presence of high intensity and phase gradients in the superoscillatory field. This stronger sensitivity of the superoscillatory field explains the enhancement of resolution comparatively to plane wave illumination. In particular (see Figure 5c), placing the particle anywhere apart from the very narrow subwavelength singularity zone (black horizontally extended area indicated by green dotted line) results in a strong change of intensity across the detector plane. Figure 5d shows that when the nanoparticle is repositioned away from the singularity point in the object plane, a very narrow, deeply subwavelength zone is created on the detector plane where no change of intensity is taking place. These features can be used to accurately retrieve the particle position. We argue that enhancement of resolution with topological light against plane wave illumination shall increase with the number of positions at which the object is illuminated. Indeed, fine scanning of the object in the topological field improves chances of overlap between the fine features of the object and zones of rapid phase variation that are crucial for high resolution image reconstruction.

The deeply subwavelength topological microscopy (DSTM) reported here shall be compared with confocal microscopy that uses a superoscillatory subwavelength intensity hotspot for object illumination.^[^ ^25^, ^26^ ^]^ The image is reconstructed point‐by‐point by scanning the hotspot against the object while it is imaged by a conventional lens through a confocal aperture. In this case, the size of the superoscillatory hotspot determines the resolution of the technique.^[^ ^27^ ^]^ Although, in principle, the superoscillatory hotspot can be arbitrary small, intensity in the hotspot rapidly drops with its size, and resolution better than *λ*/4 is difficult to achieve. As we have shown here, the resolution of DSTM could be orders of magnitude better than confocal microscopy with superoscillatory hotspot illumination.

## 4 Conclusions

In conclusion, we have introduced and demonstrated the new concept of deeply subwavelength topological microscopy (DSTM), which employs artificial intelligence to retrieve, with deeply subwavelength resolving power, dimensions of a physical object from its scattering pattern upon topological illumination. Although so far DSTM has been demonstrated for 1D imaging, we argue that it could be readily extended to 2D and 3D objects, as well as objects of random shape, and could be very efficient in microscopy of a priori known shapes, such as found in routine tasks of machine vision, smart manufacturing, particle counting for life sciences application, etc. The technique does not require labelling of the sample with luminescent materials, nor intense laser illumination thus avoiding photodamage effects, is resilient to noise and we expect that it can work in both transmission and reflection mode depending on the imaging target. The technique could lead to far‐reaching consequences across a number of disciplines, such as biomedical sciences, materials science and nanotechnology.

## Conflict of Interest

The authors declare no conflict of interest.

## Supporting information

Supporting Information
