# Supplementary material for: Unlabeled Far‐Field Deeply Subwavelength Topological Microscopy (DSTM)
Source: Adv Sci (Weinh). 2020 Nov 17;8(1):2002886. doi: 10.1002/advs.202002886 (PMC7788582; doi:10.1002/advs.202002886)
Supplement: Supplementary file 1 — Supporting Information [file ADVS-8-2002886-s001.pdf]

© 2020 Wiley-VCH GmbH

ADVANCED SCIENCE
Open Access

Supporting Information

for *Adv. Sci.,* DOI: 10.1002/advs.202002886

Unlabeled Far-Field Deeply Subwavelength Topological Microscopy (DSTM)

*Tanchao Pu, Jun-Yu Ou, Vassili Savinov, Guanghui Yuan, Nikitas Papasimakis, and Nikolay I. Zheludev**

Supporting Information

Unlabeled Far-field Deeply Subwavelength Topological Microscopy

*Tanchao Pu, Jun-Yu Ou, Vassili Savinov, Guanghui Yuan, Nikitas Papasimakis, Nikolay I. Zheludev**

S1. Numerical methods

In the numerical imaging experiments, the superoscillatory field generator is scanned across the object by steadily moving the superoscillatory hotspot, at intervals of $\lambda/5$, from the -λ position to the +λ position in the object plane. For each position of the hotspot, the detector records a diffraction pattern. The full set of diffraction patterns is analyzed by a Convolutional Neural Network[1] to retrieve information about the object. The network contains three convolution layers with 32-5×5, 32-3×3, 64-3×3 and 32-1×1 kernels, correspondingly, and three fully connected layers with 128, 32, 4 neurons, respectively. Each of the first three convolution layers is followed by a pooling layer with 1×4, 1×8, 1×4 kernels with Rectified Linear Unit activation function. The network was trained with the Adam stochastic optimization method[2] and mean absolute error loss function, aimed at improving the retrieval of the dimer geometrical dimensions, i.e. constants *A*, *B*, *C* and *D*. The training dataset contained 20,000 samples and was generated by creating dimers of random sizes and placing them on the object plane with the dimer centre coordinate *D* randomly chosen in the interval from $-\lambda/2$ to $\lambda/2$ (in the case of unknown dimer position). The widths of the dimer components (*A* and *C*) and the gap between them (*B*) were independently and randomly chosen between 0.002λ and λ. The diffraction pattern on the detector array was then calculated by the Fourier propagation method[3] for the transverse component of the electric field.

In the real-life imaging experiments with superoscillatory illumination, the recorded scattering patterns were processed by a convolutional neural network consisting of 4

convolutional layers, 2 dense layers activated by a ReLU function, and an output layer with a sigmoid activation function. The first three convolutional layers contained 64, 112, and 112 3*3-filters, while the fourth convolutional layer contained 32 1*1-filters. There were 64, 16 and 3 neurons in the dense layers and output layer, respectively. In the case of plane wave illumination, the neural network comprised 3 dense layers with 128, 512, 256 neurons activated by ReLU function and an output layer with 3 neurons activated by a sigmoid function.

The training time in the case of the numerical experiments was ~1h using a multi-GPU server (4 x GTX1080), while for the experimental results training took ~2 min in a standard desktop PC.

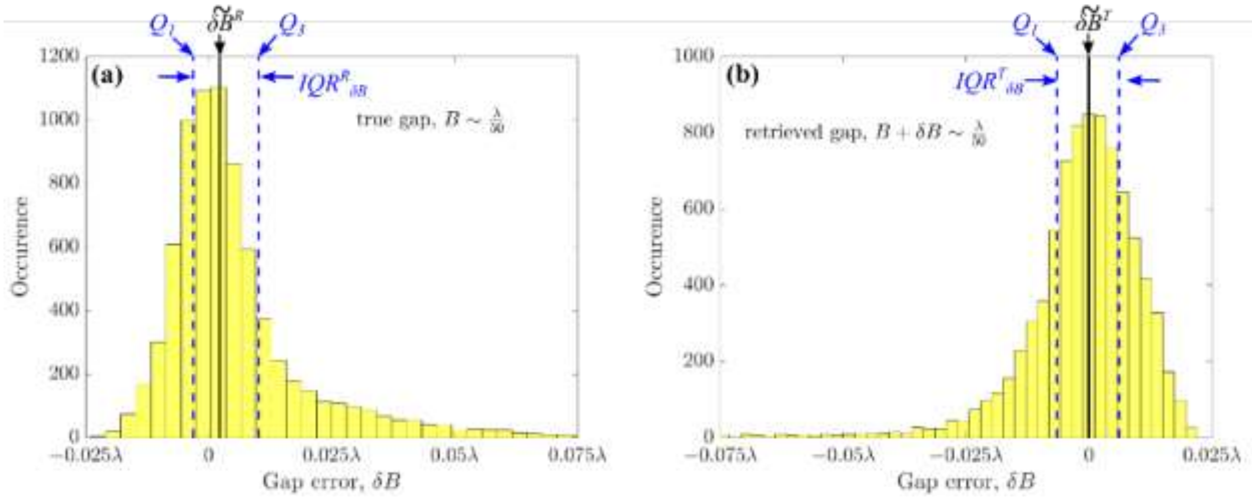

Figure S1. Evaluation of DSTM resolution. Panels (a) and (b) illustrate the two different ways in which errors of measuring the dimer gap *B* can be defined, namely given a true parameter value (panel (a)) or a retrieved parameter value (panel (b)). The measurement events are placed in the bins of corresponding true value in panel (a) and corresponding retrieved value in panel (b). The panels show histograms of characteristic distributions of errors for *B* ~ *λ*/50. The solid black line marks the median ($\widetilde{\delta B}^R$,) of the error distribution. The interquartile ranges $IQR^R{}_{\delta B}$ and $IQR^T{}_{\delta B}$ are defined as the ranges between the corresponding first and third quartiles, which include 50% of the error values.

S2. Statistical analysis and resolution of DSTM

We quantify the error in the estimation of each of the four geometric parameters of the dimer *(A, B, C, D)* by imaging a large test set consisting of ∼7×10^5 dimers with random parameters. The dimer parameters are selected in the range $\lambda/100<A,B,C<\lambda$ and $-\lambda/2<D<\lambda/2$ according to a random uniform distribution. We consider two closely related but distinct approaches of defining errors of the retrieval process, see Figure S1. In both cases, a retrieval event is represented by the true gap value of the measured parameter, e.g. *B*, and its retrieved value $B+\delta B$.

In the first approach, we examine the spread of measured values of the dimer parameter for a given true value of the parameter**.** Here we define bins for true values and examine the distribution of retrieved values, $B+\delta B$, and corresponding errors, δB, within each bin. An example of the distribution is presented in Figure S1a where the true gap value *B* is around λ/50 ($0.01925\lambda<B<0.02075\lambda$). The distribution of the errors for the retrieval attempts can be characterized by the median value $\widetilde{\delta B}^R$ and the corresponding interquartile range $IQR^R_{\delta B}$, defined as the range between the second and third quartiles of the distribution containing 50% of all attempts. This procedure is repeated for all bins of true gap size, *B*. A similar procedure is followed for the dimer parameters *A*, *C* and *D*. In the second approach, we examine the spread of true values of the dimer parameter for a given retrieved value of this parameter. Here, we define the bins along with the retrieved values and examine the distribution of true values, *B*, and corresponding errors, δB, within each bin. The distribution is characterized by the median $\widetilde{\delta B}^T$ and $IQR^T_{\delta B}$, see Figure S1b. The corresponding resolution is defined as $IQR^T_{\delta B}/2$. Again, a similar procedure is followed for the dimer parameters A, C and D.

Medians and *IQR*s calculated according to the first approach are presented in Figure S2, while the results presented in the main text are calculated according to the second approach, see Figure 2-3 in the main text. From these graphs, one can see that both approaches return similar systematic offsets and resolution powers.

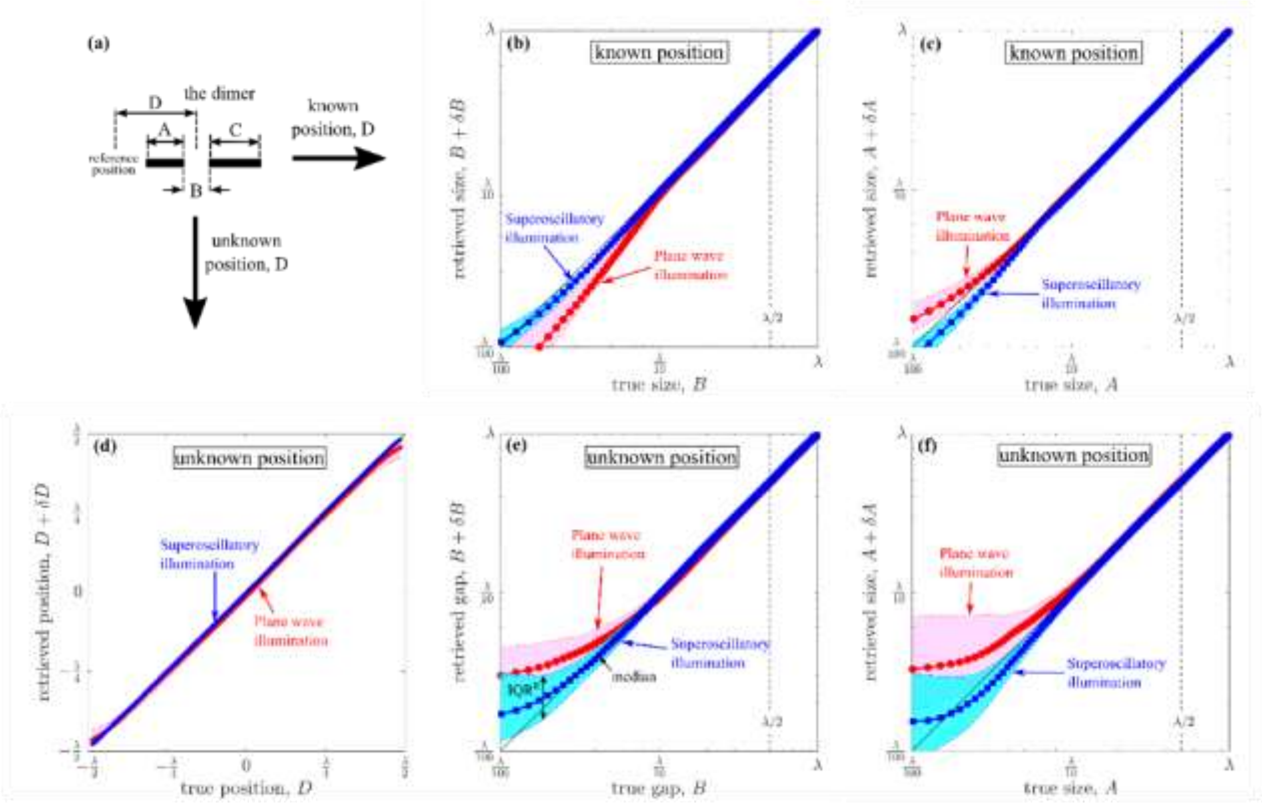

Figure S2. Imaging errors for dimer imaging. The retrieved values of the dimer parameter for a given true value of the parameters are presented**.** (a) Schematic of the dimer and its geometric parameters. Panels (b) and (c) show measurement results if location *D* of the dimer is known. Solid blue and red lines correspond to the median of the retrieved values under superoscillatory (blue squares) and plane wave illumination (red circles), while the red and blue colored bands indicate the corresponding interquartile (*IQR*) ranges. In the case of the unknown position of the dimer, panels (d-f) show the retrieved values of *A*, *B* and *D* against their actual values. Median and *IQR* values are calculated over bins containing 5,000 dimers each according to the first approach as described in the Supporting Information.

S3. DSTM through noise

Here we examine sensitivity of the DSTM method to noise. In a practical implementation of the method, noise can arise either as detection noise or due to unwanted scattering and interference effects. We model both of these effects by introducing an effective "noise field", E_n, at the detector plane, which takes values according to a zero-mean Gaussian distribution with standard deviation σ.

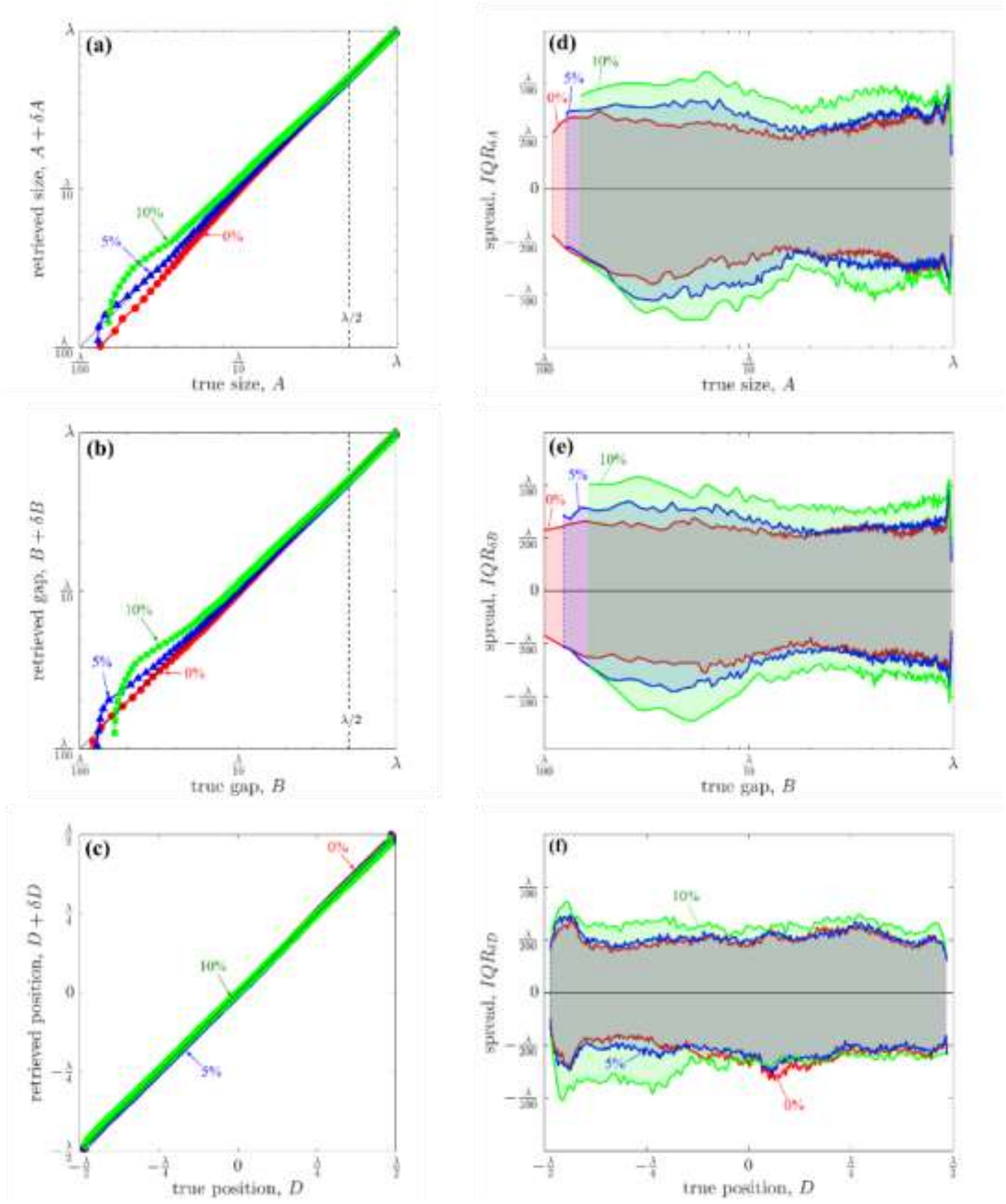

Fig S3. Resilience of DSTM to noise. The figure shows the effects of different levels of noise η on the retrieved dimer element size *A* (a,b), gap *B* (b,e), and position *D* (c,f). Panels (a-c)

show the median of retrieved values for η = 0% (red circles), 5% (blue triangles) and 10% (green squares). Red, blue, and green colored regions in panels (d), (e) and (f) show the spread (IQR) of errors for η = 0%, 5%, and 10%, respectively. Median and IQR values are calculated over bins containing 5,000 dimers each according to the second approach as described in the Supporting Information section S1.

Thus, in the scenario of imaging a dimer, the total electric field at the detector plane will be the sum of the field, E_s, scattered by the dimer and the effective noise field, E_n. The corresponding total light intensity will be: $I = |E_s + E_n|^2$, or equivalently $I = |E_s|^2 + |E_n|^2 + 2Re[E_s E_n{}^*]$. The first term in this equation is the intensity of the field scattered by the dimer, the second term represents the detector's noise, while the third term accounts for the interference effects between light scattered from the dimer and any unwanted scattering. Assuming $E_s \gg E_n$, we quantify the noise level by the ratio $\eta = \frac{\sigma}{\max(|E_s|)}$, where $\max(|E_s|)$ is the maximum value of the modulus of the electric field at the detector plane.

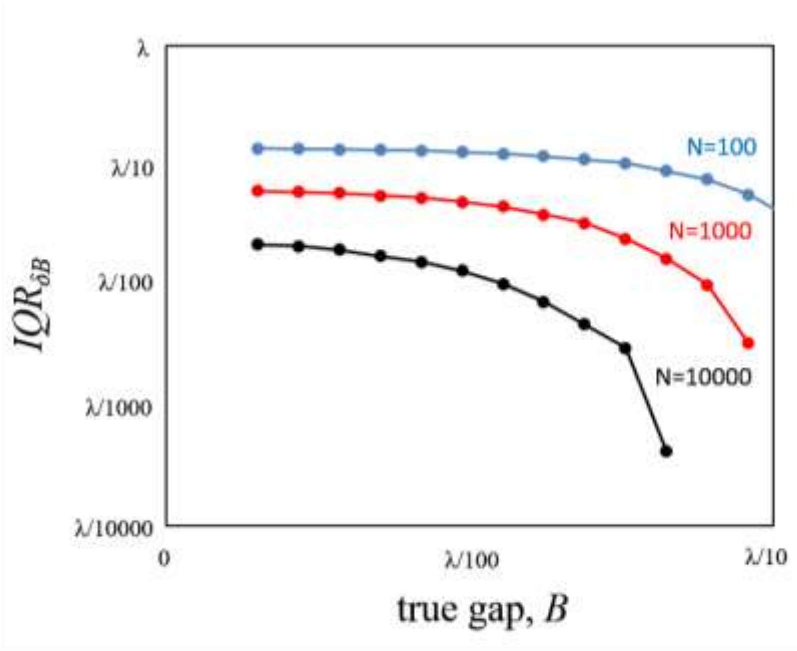

Fig S4. Dependence of resolution on training dataset size. The figure shows the resolution of DSTM for the dimer gap, B, for dataset comprising scattering events on 100 (blue), 1,000 (red), and 10,000 (blue) dimers under superoscillatory illumination.

Figure S3 shows the effect of noise on the retrieval of the dimer geometrical parameters. The noise results in the increase of divergence (bias) of the median lines (Figure S3a&b) from the line of perfect imaging (black line in Figure S3). In the case of 5% noise, substantial divergence occurs for $A<\lambda/77$ and $B<\lambda/65$, while for noise levels of 10%, the median diverges for $A<\lambda/45$ and $B<\lambda/60$.

The effects of noise on resolving power are presented in Figure S3d-f. Here, increasing the noise level leads to gradual decrease of resolution. However, for all measured parameters resolution at 5% and 10% noise level remains at deeply subwavelength level, i.e. better than ~λ/70 and ~λ/55 correspondingly. This illustrates a remarkable resilience of the deconvolution process considering that we account for the interference phase related effects in the noise without providing any phase information to the network.

Table S1. Resolution under superoscillatory and plane wave illumination. The values in square brackets (for unknown position and under superoscillatory illumination) correspond to the resolution in the presence of 5% noise.

	**Superoscillatory illumination**		**Plane wave illumination**	
	Unknown position	Known position	Unknown position	Known position
**Resolution in dimer element size, *A***	0.0133λ (λ/75) [0.0142λ (λ/71)]	0.0045λ (λ/222)	0.0214λ (λ/47)	0.0064λ (λ/156)
**Resolution in dimer gap, *B***	0.0122λ (λ/82) [0.0130λ (λ/77)]	0.0042λ (λ/238)	0.0192λ (λ/52)	0.0061λ (λ/164)
**Resolution in position, *D***	0.0111λ (λ/90) [0.0108λ (λ/92)]		0.0297λ (λ/34)	

S4. Experimental setup for DSTM

The experimental demonstration of DSTM was carried out in a dual microscope with a custom built computer-controlled wavefront synthesizer system based on spatial light modulators (Meadowlark P512) and coherent laser source at wavelength λ=488nm (Newport Excelsior-

ONE 488nm, 100mW) (see Fig. S5) [5]. The illumination light field at focus is optimized with respect to the desired field through an iterative process [5]. We note that compared to metasurface-based schemes of superoscillatory light field generation, SLMs provide dynamic control of the illumination of the light field at the cost of stability.

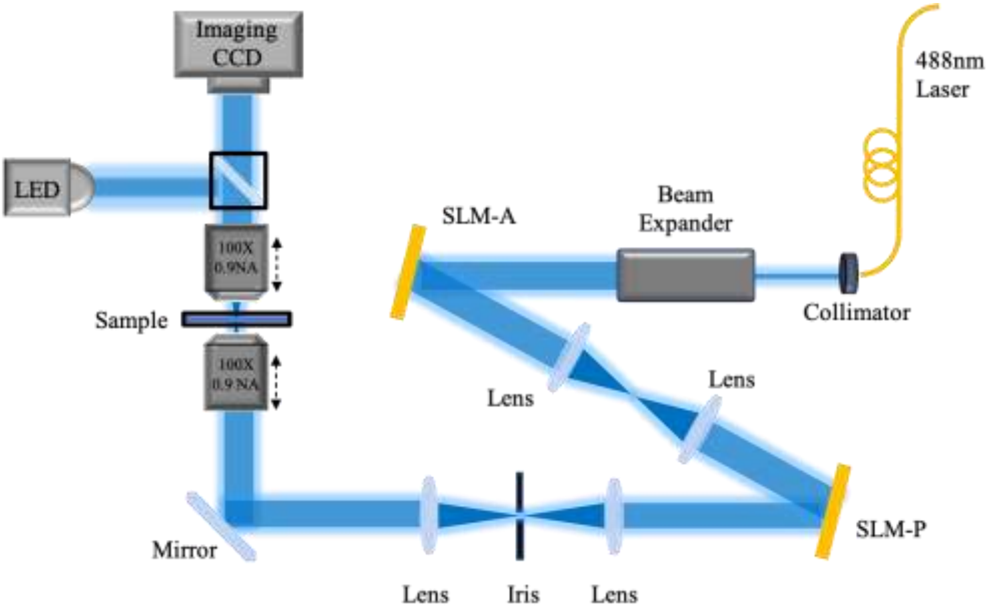

Fig S5. Experimental setup for DSTM. SLM-A – Spatial light modulator used in amplitude modulation mode, SLM-P – Spatial light modulator used in phase modulation mode.

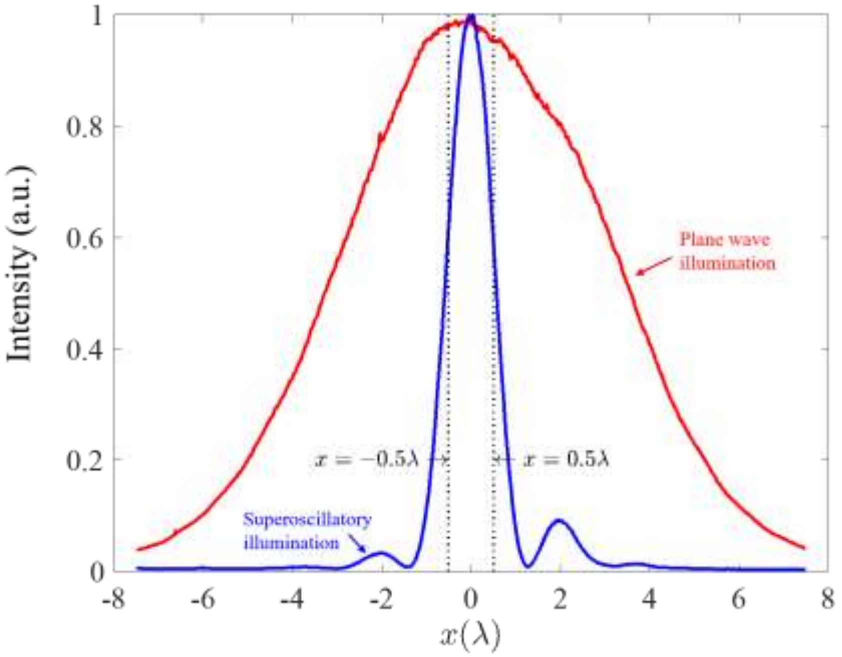

Fig S6. Plane wave (red) and superoscillatory (blue) illumination profiles in the experimental implementation of DSTM. The field profiles have been imaged at the focus. The corresponding

focal spot sizes are ~6.7λ and ~1.1λ for plane wave and superoscillatory illumination, respectively. For both types of illuminations, the wavelength is λ=488 nm.

The sample was placed at the focus of the illumination light field and the resulting diffraction pattern was collected by a high NA objective and a CCD array. Two different types of illumination were considered, topologically structured illumination and Gaussian illumination (see Fig. S6). Typical examples of recorded diffraction patterns under superoscillatory and Gaussian illumination are shown in Figure S7. The acquisition time of each diffraction pattern was ~1s. The 2D recorded patterns are transformed into 1D traces by considering only the central part of the diffraction pattern along the slit.

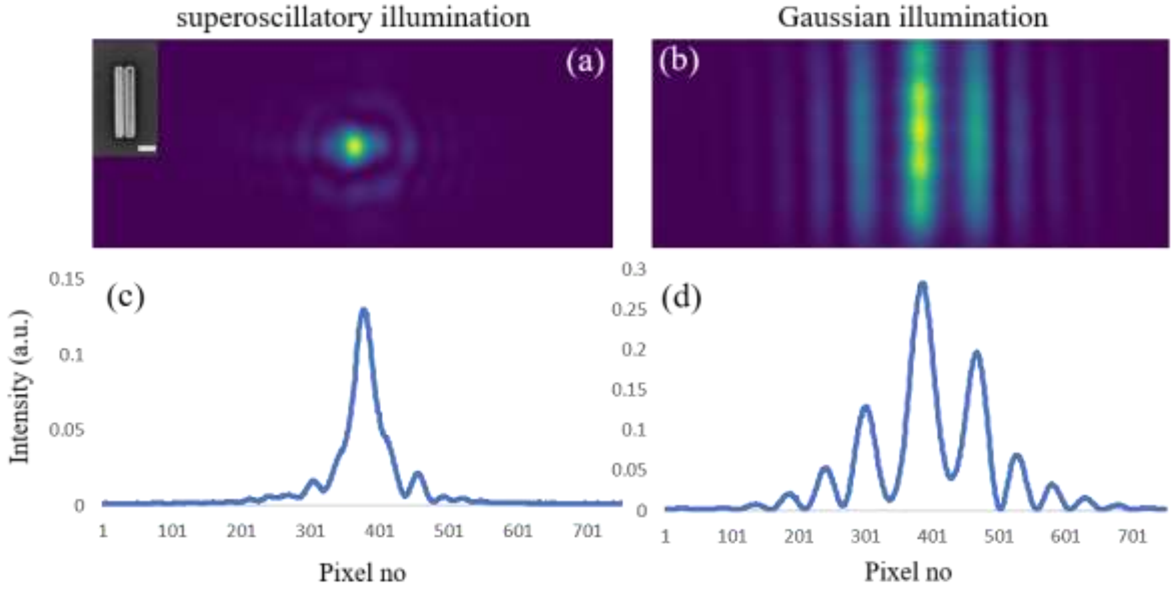

Fig S7. Characteristic experimentally recorded 2D diffraction patterns (a-b) and extracted 1D traces (c-d) under illumination with superoscillatory (a,c) and Gaussian profile (b,d) light fields. The inset to (a) shows an SEM image of the imaged dimer. Scale bar is 1 μm.

References

[1] A. Krizhevsky, I. Sutskever, G. E. Hinton, *Adv. Neural Inf. Process. Syst.* **2012**, *25*, 1106.

[2] D. Kingma, J. Ba, *arXiv:1412.6980* [cs.LG], **2014**.

[3] J. W. Goodman, *Introduction to Fourier optics*, W.H. Freeman, Macmillan Learning, New York, USA **2017**.

[4] G. H. Yuan, K. S. Rogers, E. T. F. Rogers, N. I. Zheludev, *Phys. Rev. Applied* **2019**, *11*, 064016.

[5] E. T. F. Rogers, S. Quraishe, K. S. Rogers, T. A. Newman, P. J. S. Smith and N. I. Zheludev. *APL Photonics* **2020**, 5, 066107.
